# Deposition of Pd, Pt, and PdPt Nanoparticles on TiO_2_ Powder Using Supercritical Fluid Reactive Deposition: Application in the Direct Synthesis of H_2_O_2_

**DOI:** 10.3390/molecules29092142

**Published:** 2024-05-05

**Authors:** Marlene Crone, Laura L. Trinkies, Roland Dittmeyer, Michael Türk

**Affiliations:** 1 Institute for Technical Thermodynamics and Refrigeration, Karlsruhe Institute of Technology (KIT), Engler-Bunte-Ring 21, 76131 Karlsruhe, Germany; 2 Institute for Micro Process Engineering, Karlsruhe Institute of Technology (KIT), Hermann-von-Helmholtz-Platz 1, 76344 Eggenstein-Leopoldshafen, Germany

**Keywords:** SFRD, Pd, Pt, PdPt nanoparticles, TiO_2_, H_2_O_2_ direct synthesis

## Abstract

In this study, we investigated the catalytic properties of mono- and bimetallic palladium (Pd) and platinum (Pt) nanoparticles deposited via supercritical fluid reactive deposition (SFRD) on titanium dioxide (TiO_2_) powder. Transmission electron microscopy analyses verified that SFRD experiments performed at 353 K and 15.6 MPa enabled the deposition of uniform mono- and bimetallic nanoparticles smaller than 3 nm on TiO_2_. Electron-dispersive X-ray spectroscopy demonstrated the formation of alloy-type structures for the bimetallic PdPt nanoparticles. H_2_O_2_ is an excellent oxidizing reagent for the production of fine and bulk chemicals. However, until today, the design and preparation of catalysts with high H_2_O_2_ selectivity and productivity remain a great challenge. The focus of this study was on answering the questions of (a) whether the catalysts produced are suitable for the direct synthesis of hydrogen peroxide (H_2_O_2_) in the liquid phase and (b) how the metal type affects the catalytic properties. It was found that the metal type (Pd or Pt) influenced the catalytic performance strongly; the mean productivity of the mono- and bimetallic catalysts decreased in the following order: Pd > PdPt > Pt. Furthermore, all catalysts prepared by SFRD showed a significantly higher mean productivity compared to the catalyst prepared by incipient wetness impregnation.

## 1. Introduction

Supercritical fluid-based particle formation technologies are increasingly being used to develop novel functional nanostructured materials or to improve the properties of existing ones. Such supported metal or metal oxide nanoparticles (NPs) are frequently used in catalysis, electronics, optics, and sensing [1]. The supported materials can exist in different morphologies such as atomically dispersed metals, NPs, nanorods, or thin films. Especially, supported NPs are of particular scientific interest and have a high potential for use in a variety of technical applications due to their higher surface-to-volume ratios. For example, the use of supported noble metal catalysts for hydrogenation and/or oxidation processes is common in basic research and industrial applications.

Catalysis is a particularly important application for supported NPs, since roughly 90% of the currently employed chemical processes and thus the majority of all goods produced are based on catalytic reactions [2,3]. Catalysts are generally metal or metal oxide NPs with sizes from 1 to 10 nm and outstanding surface-to-volume ratios. Thereby, it is worthy to consider that a decrease in particle size (PS) from 10 to 1 nm will result in an increase in the amount of shell atoms from 20% to 99% and that these surface atoms are chemically more active compared to the bulk atoms [4]. To understand the relationship between PS and catalytic activity, it must be considered that, as a rule of thumb for monometallic NPs, the catalytic activity depends on the fraction of surface atoms on corners and edges, i.e., the PS, while for bimetallic NPs, the catalytic productivity is also strongly influenced by surface segregation [5].

Metal catalysts are of considerable interest and have been successfully implemented for many tasks, whereby Pd- and Pt-based NPs are being widely studied owing to their versatile application profile. The deposition of NPs on porous substrates can be achieved using a variety of conventional and established techniques, e.g., incipient wetness impregnation (IWI), co-precipitation (CP), chemical vapor deposition (CVD), and flame spray pyrolysis (FSP). However, these methods have disadvantages and limitations such as the requirement of liquid reactant solutions, energy-intensive drying steps, broad particle size distributions (PSD), extremely low sublimation pressures of the metal precursors, high processing temperatures, and frequently, also mass transfer-limited kinetics. A further disadvantage of these techniques is the fact that they cannot be easily applied to aerogels and to polymeric substrates due to the high temperatures that are required to reduce the precursors.

The application of the SFRD process and, especially, the use of supercritical carbon dioxide (scCO_2_) as the process medium for the preparation of catalysts offer advantages over the above-mentioned conventional catalyst preparation methods. CO_2_ is inexpensive, inert, non-flammable, and non-toxic; moreover, it has moderate critical temperature and pressure (*T_c_* = 304.2 K, *p_c_* = 7.4 MPa) [6]. Due to the liquid-like density of CO_2_ in the supercritical state, the dissolved metal precursor concentration can be up to three orders of magnitude higher than in CVD. In addition, the high binary diffusion coefficient *D*_12_ leads to an enhanced mass transfer rate and therewith promotes a fast penetration into pores. Likewise, the gas-like low viscosity and lack of surface tension of scCO_2_ promote an effective impregnation of complex pore architectures and confined geometries.

It has to be mentioned that the SFRD process is identical to the so-called chemical fluid deposition (CFD), supercritical fluid deposition (SCFD), supercritical deposition (SCD), and supercritical fluid chemical deposition (SFCD) techniques. In fact, the basic principles of the deposition and particle formation process are the same. In general, two approaches are commonly employed: a thermodynamically controlled process and a kinetically controlled process. The latter approach was developed by the group of Aymonier as a fast and versatile process for kinetically controlled surface structuration. More details about this promising method and the obtained results can be found in the literature [7,8,9]. Detailed surveys of the results obtained from a large number of different thermodynamically controlled SFRD experiments were provided by Siril and Türk [10] and recently by Yousefzadeh et al. [11]. A comprehensive compilation of the studies published in the literature on the preparation of supported nanoparticles on various substrates by SFRD is presented in chapter 7 in [12].

**Figure 1:**
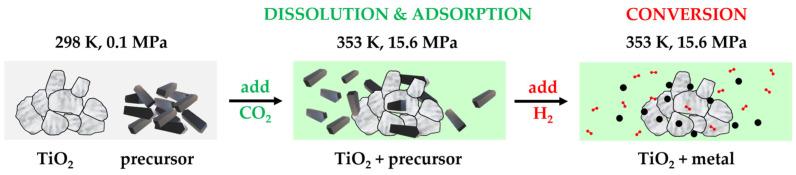
Principle of the synthesis of supported NPs by SFRD using scCO_2_ as a solvent and as a reaction and separation medium.

In brief, the SFRD process for depositing metal NPs onto the surface of a porous substrate involves the three steps described and illustrated in Figure 1. First of all, the precursor is dissolved in scCO_2_, which is followed by the molecular adsorption of the precursor on the substrate. Finally, the precursor is converted to its metal form by adding hydrogen (H_2_). Due to the complete miscibility of scCO_2_ with reacting gases such as H_2_ as well as with the conversion products, CO_2_ acts as a solvent and as a reaction and separation medium in the SFRD process. Since no liquid byproducts are generated, a solvent-free and dry product is obtained in an integrated process.

In the SFRD process, the PS and PSD can be influenced by the reduction method and conditions, the properties of the substrate (surface and chemical nature), the type and amount of precursor in the system, and thus, the solubility of the precursor and its adsorption behavior [11]. A detailed overview about the influence of the precursor solubility and adsorption behavior on the PS can be found in a review article published recently [13].

From a more general perspective and based on the results of a large number of gas-phase experiments on CO and/or NO oxidation, it was shown that the SFRD technique leads to catalysts with higher catalytic activity compared to conventional preparation methods. In opposite thereto, there are only a very limited number of investigations dealing with heterogeneously catalyzed reactions in the liquid phase such as the direct synthesis of H_2_O_2_ in an aqueous medium.

H_2_O_2_ is increasingly gaining significance as a so-called green oxidizing agent. Being highly active, H_2_O_2_ is receiving more and more attention in sectors such as the paper and pulp industries or waste water treatment [14,15,16]. Recently, due to the COVID-19 pandemic, H_2_O_2_ has been gaining relevance in disinfection applications, such as the reprocessing of clinical masks [17,18]. However, due to the high risks and costs associated with the transport of highly concentrated H_2_O_2_, it is desirable to replace the mostly centrally organized H_2_O_2_ production plants with a decentralized production system [19,20]. An approach that has attracted increasing interest is the direct synthesis of H_2_O_2_ from H_2_ and O_2_ in a liquid solvent. Particular attention is being paid in this area to the synthesis of suitable catalysts with high H_2_O_2_ selectivity and productivity [21,22].

This paper is organized as follows. We report the results of SFRD experiments on the deposition of mono- and bimetallic Pd and Pt NPs on TiO_2_ powder. Based on this, we discuss the influence of the different metallic NPs on the direct synthesis and production of H_2_O_2_ and compare their productivities with that of a catalyst synthesized by the conventional IWI process. Afterwards, a description of the experimental setup and of the execution of the experiments and a data evaluation are presented.

## 2. Results and Discussion

The aimed metal loading of 1 wt% was proved via inductively coupled plasma optical emission spectrometry (ICP-OES). The following acronyms will be used in the subsequent discussion: CAT-Pd1 stands for the catalyst obtained from palladium (II) acetylacetonate (Pd(acac)_2_), CAT-Pd2 was obtained from palladium (II) bis(2,2,6,6-tetramethyl-3,5-heptanedionato) (Pd(tmhd)_2_), CAT-Pt was obtained from platinum (II) dimethyl(1,5-cyclooctadiene) (Pt(cod)me_2_), and CAT-Pd1Pt was obtained from a mixture of Pd(acac)_2_ and Pt(cod)me_2_.

For comparison with the catalysts prepared by SFRD, a catalyst produced by means of IWI was used (CAT-Pd3-IWI). Thereby, TiO_2_ was impregnated with an aqueous solution containing palladium (II) nitrate hydrate (Pd(NO_3_)_2_ · H_2_O). More details about the procedure can be found in the literature [23,24].

### 2.1. Size and Metal Loading of the Catalysts

**Figure 2:**
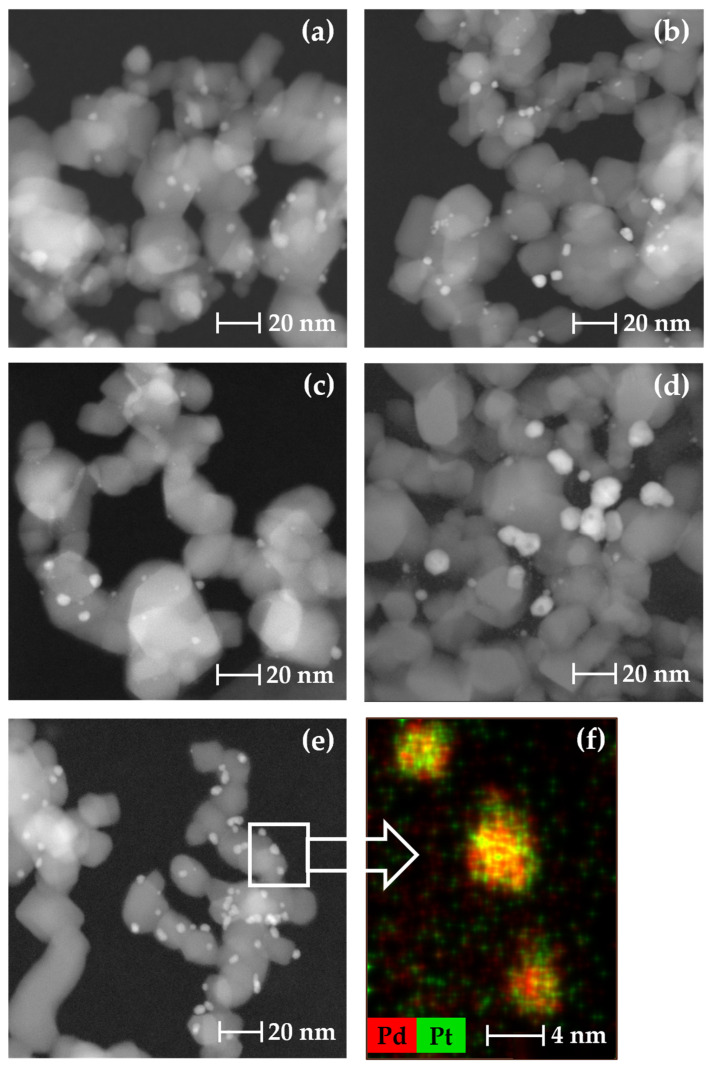
Representative high-angle annular dark-field TEM images of CAT-Pd1 (**a**), CAT-Pt (**b**), CAT-Pd2 (**c**), CAT-Pd3-IWI (**d**), and CAT-Pd1Pt (**e**) and the corresponding EDXS image (**f**).

The size of the metal NPs of the catalysts synthesized by SFRD and IWI was characterized using transmission electron microscopy (TEM) images, while the structure of the bimetallic catalyst was determined additionally by electron-dispersive X-ray spectroscopy (EDXS). High-angle annular dark-field TEM images of the different monometallic NPs deposited on TiO_2_ are presented in Figure 2a–d, and a typical TEM image of bimetallic Pd1Pt NPs with the corresponding EDXS mapping image is shown in Figure 2e,f. The mean PS *x*_50_ is defined as the diameter size that indicates that 50% of the particles have a larger diameter, and the other 50% have a smaller diameter; PS *x*_10_ and PS *x*_90_ are defined accordingly. The width of the PSD, *Δ*, is defined as *Δ* = [(*x*_90_ − *x*_10_)/2 · *x*_50_], which describes the polydispersity of the various samples. The resulting PS (*x*_10_, *x*_50_, and *x*_90_) and PSD *Δ* values of the metal NPs synthesized either by SFRD or IWI, along with the corresponding metal loadings, determined by ICP-OES, of the different catalysts and the mean productivities for the different catalysts are summarized in Table 1. The EDXS mapping images such as those in Figure 2f indicated that the CAT-Pd1Pt sample showed a uniform distribution of Pd and Pt and, in agreement with the ICP-OES results (see Table 1), about equal amounts of Pd and Pt. Furthermore, the EDXS mapping indicated the formation of alloy-type structures for the bimetallic Pd1Pt nanoparticles.

**Figure 3:**
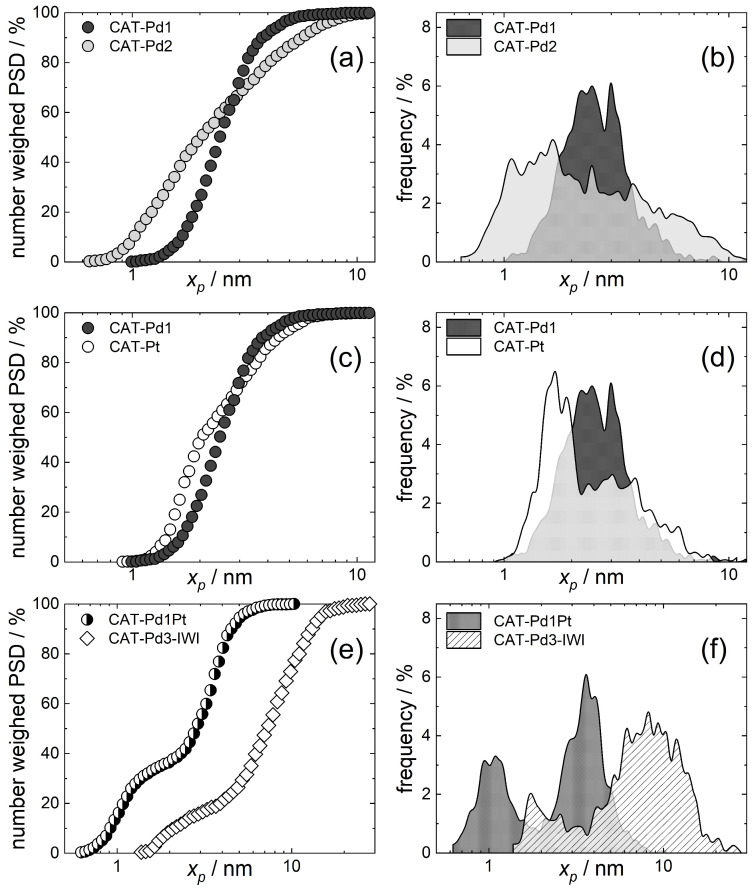
PSD and PS frequencies of Pd1 and Pd2 (**a**,**b**), Pd1 and Pt (**c**,**d**), and Pd1Pt and Pd3-IWI (**e**,**f**).

Figure 3 shows the number-weighted PSDs and the PS frequencies of the examined samples derived by particle counting. These diagrams show that small mono- and bimetallic Pd, Pt, and PdPt NPs with mean particle sizes *x*_50_ ranging from 2.0 to 2.8 nm and particle size distributions *Δ* ranging from 0.43 to 1.16 were deposited on TiO_2_. The mean PS increased in the order CAT-Pd2 = CAT-Pt < CAT-Pd1 < CAT-Pd1Pt, while *Δ* increased in the order CAT-Pd1 < CAT-Pd1Pt < CAT-Pt < CAT-Pd2. Interestingly, the substitution of (acac)_2_ with the (tmhd)_2_ ligand led to smaller Pd particles of 2.0 nm instead of 2.5 nm but a significantly broader PSD *Δ* of 1.16 compared to 0.43. Among other reasons, this might have been caused by the different adsorption behaviors of the different ligands. The interpretation of this result is the subject of ongoing and future investigations.

As depicted in Figure 3c,d, the Pt NPs were smaller than the Pd1 NPs (2.0 nm vs. 2.5 nm) but showed a significantly broader PSD *Δ* of 0.74 compared to that of 0.43 of the latter. Finally, from Table 1 and Figure 3 it follows that in the case of the bimetallic Pd1Pt NPs (wt% = 0.51/0.49), a bimodal PSD with a narrow *Δ* = 0.64 was obtained. In opposite thereto, significantly larger nanoparticles with a mean particle size of 7.2 nm, a bimodal PSD, and a *Δ* = 0.76 were obtained for CAT-Pd3-IWI.

Until today, only limited studies on the formation of bimetallic NPs via SFRD have been published in the literature, and a brief overview of them is provided below. Among other bimetallic NPs, PdPt NPs were deposited on multi-walled carbon nanotubes via simultaneous SFRD with scCO_2_ by Yen et al. [25]. In these experiments, the deposition temperature was 473 K, and the conversion of the precursors was performed by chemical reduction with H_2_ at 25 MPa, resulting in bimetallic NPs with a mean PS of 9.2 nm. Experiments performed at 323 K and 15 MPa by Qiao et al. showed that PdPt NPs with a mean PS of 5.0 nm were deposited on SBA-15 via SFRD and resulted in a superior activity compared to catalysts prepared with the common liquid impregnation method using conventional liquid solvents [26]. Erkey and coworkers deposited PdPt NPs on Black Pearls^®^ by simultaneous and sequential deposition [27,28]. Particles of similar shape and size were synthesized at 353 K and 27.6 MPa, and thereby a mean PS of 4.3 nm was obtained by simultaneous deposition. In opposite thereto, sequential deposition led to particles with sizes between 5 and 15 nm. Hu et al. deposited Pd, Pt, and PdPt NPs on porous carbon [29]. Thereby, a number-averaged diameter of 4.7 nm was obtained for the bimetallic PdPt NPs.

To our knowledge, bimetallic PdPt NPs on TiO_2_ have not yet been synthesized by means of the SFRD process. It should be noted that the synthesis of other bimetallic particles such as, e.g., PtAu, PtCu, PtNi, and PtRu [25,30,31,32] is not the subject of this paper and was beyond the scope of this paper.

### 2.2. Productivity of the Different Catalysts

The productivities of TiO_2_-supported Pd, Pt, and PdPt NPs in the direct synthesis of H_2_O_2_ were investigated in a continuous-flow reactor at ambient temperature and pressure in an aqueous reaction medium, and the results are summarized in Table 1.

**Figure 4:**
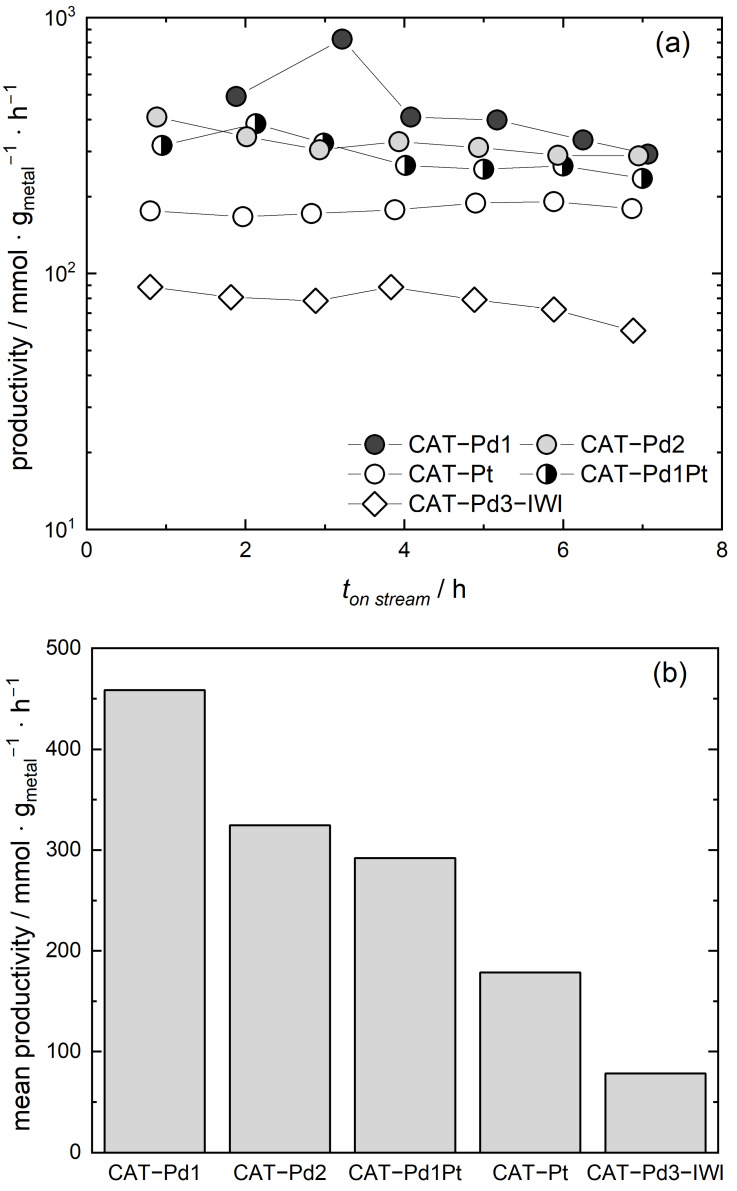
Productivities calculated and plotted over time for the different catalysts investigated (**a**) and mean productivities for the different catalysts evaluated (**b**).

Figure 4a shows the productivities calculated from the measured concentrations of H_2_O_2_ for the catalysts investigated vs. the time of the experimental run, whereas Figure 4b shows the resulting mean productivities. Note that, to highlight the differences in the mean productivities, the *y*-axis values in Figure 4b are plotted linearly. As it can be seen in both graphs, CAT-Pd-1 led to the highest productivity, while CAT-Pt-1 led to the lowest productivity among the catalysts produced by SFRD; however, these productivities were still significantly higher than that measured for CAT-Pd3-IWI. Thus, the mean productivities decreased in the following order: CAT-Pd1 > CAT-Pd2 > CAT-Pd1Pt > CAT-Pt > CAT-Pd3-IWI. Although CAT-Pd2 had a smaller PS (2.0 nm) than CAT-Pd1 (2.5 nm), the average productivity for CAT-Pd2 was lower, which is consistent with findings regarding CO oxidation [33]. One further explanation of the differences in catalytic productivity relates to the different values of the respective PSD *Δ*, which was significantly broader for CAT-Pd2 (1.16) compared to CAT-Pd1 (0.43).

Beyond that, a stronger influence of the metal (Pd or Pt) than of the ligands ((acac)_2_ or (tmhd)_2_) on the mean productivity of the various catalysts was observed. These results can be qualitatively explained by the following considerations. It should be noted that the size-dependent catalytic activation energy is the energy quantity that must be overcome in order for a chemical reaction to occur in the presence of a catalyst. This means that the lower the catalytic activation energy, the more active the catalyst. Guisbiers et al. [5] showed that the size-dependent catalytic activation energy for spherical Pd-NPs is significantly lower than that for Pt-NPs (0.56 vs. 0.62, values determined for a PS of 4 nm), which is in agreement with the higher mean productivity of CAT-Pd compared to CAT-Pt. Since Pd and Pt form an ideal mixture, surface segregation (i.e., the surface enrichment of one component of a binary alloy) can be neglected, which means that in such a binary mixture, the catalytic activation energy is a linear function of the mixture composition [5]. These reflections explain the observed result that the mean productivity observed for CAT-Pd1Pt was lower than that for CAT-Pd1 and CAT-Pd2, but higher than that for CAT-Pt.

Finally, we investigated the influence of the preparation method, i.e., SFRD compared to IWI, on H_2_O_2_ productivity through direct synthesis. Figure 4b shows that the catalysts CAT-Pd1 and CAT-Pd2 produced by SFRD exhibited a superior mean productivity, showing 6-fold and 4-fold increases, respectively, compared to the catalyst prepared by the conventional IWI procedure (CAT-Pd3-IWI). A similar result was obtained in a former investigation in which we proved that SFRD enabled the deposition of homogeneously distributed monometallic Pd and Pt and bimetallic PdPt NPs on TiO_2_-coated, three-dimensional, complex stainless-steel structures [34]. In brief, the comparison between the TiO_2_-powder catalysts discussed in the present manuscript and those obtained with TiO_2_-coated complex stainless-steel structures showed a mean productivity 5 to 16 times higher for the TiO_2_-powder catalysts. Similar results were achieved in exhaust gas catalysis, as briefly discussed below.

## 3. Experimental Section

### 3.1. Materials

For the preparation of the SFRD catalysts, the metal precursors Pd(acac)_2_, Pd(tmhd)_2_, and Pt(cod)me_2_ were obtained from abcr GmbH. Carbon dioxide (CO_2_) and hydrogen (H_2_) were received from Air Liquide. TiO_2_ powder (purity 99.5%) with a primary particle size < 21 nm, obtained from Sigma Aldrich, was used as a substrate for the deposition of mono- and bimetallic nanoparticles. The TiO_2_ powder used was a combination of anatase and rutile crystal structures. All substances were used without further purification.

The non-fluorinated variants Pd(acac)_2_ and Pd(tmhd)_2_ were used in this investigation, although the metal precursors with fluorinated alkyl groups often exhibit higher solubility in scCO_2_ [35,36]. In the case of, e.g., palladium (II) hexafluoroacetylacetonate (Pd(hfac)_2_), the undesired formation of a liquid phase occurred under the selected process conditions. Such behavior led to larger metal particles after reduction, a lower dispersion of the metal particles on the substrate, and thus a lower activity of the catalyst [37].

For the preparation of the IWI catalyst, Pd(NO_3_)_2_ · H_2_O was used, purchased from Acros Organic. Ethanol (C_2_H_5_OH) was obtained from VWR Chemicals, nitric acid (HNO_3_) was obtained from Carl Roth, sodium bromide (NaBr) was obtained from Merck, and sulfuric acid (H_2_SO_4_) was obtained from Sigma Aldrich. TiO_2_ powder (purity 99.5%) with a primary particle size < 21 nm, obtained from Sigma Aldrich, was used as a substrate for the deposition of Pd nanoparticles. Again, the TiO_2_ powder used was a combination of anatase and rutile crystal structures. Selected properties of the precursors used in this work are summarized in Table 2.

### 3.2. Apparatus and Procedure

**Figure 5:**
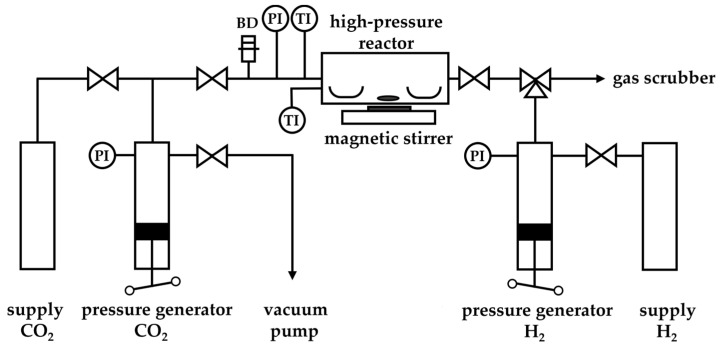
Scheme of the SFRD apparatus (BD = bursting disk, PI = pressure indicator, TI = temperature indicator).

Figure 5 depicts a scheme of the SFRD apparatus. All SFRD experiments were performed in the same process conditions of T = 353 K and *p* = 15.6 MPa. Due to their sufficient solubility in scCO_2_ in this process conditions and the fact that a solid–fluid equilibrium existed at the chosen process conditions, Pd(acac)_2_, Pd(tmhd)_2_, and Pt(cod)me_2_ were selected as precursors [38,39].

In all SFRD experiments, the metal loading was aimed at 1 wt%, and the amount of substrate was fixed to 0.210 g. Due to the different metal contents of the various precursors, the provided quantity of precursor varied from 0.007 g for Pd(acac)_2_ to 0.011 g for Pd(tmhd)_2_ and 0.004 g for Pt(cod)me_2_. Thus, in these experiments, the ratio between precursor and substrate varied between 0.02 g · g^−1^ and 0.05 g · g^−1^. Thereby, both the metal precursor and the substrate were placed into a 50 mL high-pressure reactor in two separate open recipients. For improved mixing of the supercritical solution, a magnetic stirrer was placed between the two recipients. After evacuation of the whole system at ambient temperature, the reactor was filled with CO_2_ up to 5 MPa and heated up to 353 K. When the desired process temperature was reached, the pressure was increased up to 15.6 MPa by adding scCO_2_ using a pressure generator. These conditions were held for 20 h to dissolve the precursor and to achieve the adsorption equilibrium. To initiate the conversion of the metal precursor to its metal form, an excess of H_2_ was induced into the reactor in isothermal and isobaric conditions. After a certain time for conversion (2 h), the system was slowly depressurized and cooled down to ambient conditions.

In principle, two approaches can be used for the synthesis of bimetallic NPs by SFRD: simultaneous or sequential deposition. In simultaneous SFRD, both precursors are introduced together into the high-pressure reactor and are thus simultaneously dissolved in scCO_2_ and simultaneously adsorbed onto the substrate. Subsequently, both precursors are converted to metallic NPs by means of H_2_. In sequential SFRD, the process is applied twice, introducing one precursor at a time. In the current study, bimetallic PdPt deposition was performed in a simultaneous manner. More details about the SFRD technique and the experimental procedure were published elsewhere [11]. The obtained supported metallic nanoparticles were analyzed and characterized, as described below.

### 3.3. Characterization Methods

The volumetric sorption analysis of the TiO_2_ powder was carried out with an Autosorb iQ-XR analyzer from Anton Paar (Institute of Inorganic Chemistry, IIC, Karlsruhe Institute of Technology, KIT, Karlsruhe, Germany), applying N_2_ as the adsorbate. The specific surface area was determined using the Brunauer–Emmett–Teller (BET) theory, and pore analysis was performed using density functional theory (DFT) methods. N_2_ adsorption and desorption curves confirmed the uniform mesoporous structure of TiO_2_ (BET surface area of 57 m^2^ · g^−1^, pore volume of 0.08 cm^3^ · g^−1^). For the determination of the size and size distribution of the supported mono- and bimetallic nanoparticles, TEM was used (Laboratory for Electron Microscopy, LEM, KIT, Karlsruhe, Germany). The measurements were performed using a FEI Osiris ChemiStern system (200 kV); the scanning transmission electron microscopy (STEM) images were acquired using a high-angle annular dark-field (HAADF) detector. The size of at least 1000 particles was determined statistically from the TEM images using the image analysis program ImageJ (version 1.53). Furthermore, the bimetallic NPs were analyzed by EDXS in line scans of single particles, passing through the center of the particles. ICP-OES was used to determine the metal loading. The measurements were performed with a Varian ICP-OES Vista Pro^TM^ system with a simultaneous CCD detector.

### 3.4. Determination of the H_2_O_2_ Productivity

The apparatus used in this investigation was a continuous-flow, microstructured tubular reactor [34] that was filled with the desired mass of 0.016 g of the respective catalyst, which was packed following the strategy described in the literature [40]. In brief, a pre-saturating set-up was chosen to dose the bubble-free reactant, and equimolar reactant ratios were achieved without the need of further dilution by an inert gas. The solvents were saturated with the respective reactants (H_2_ and O_2_), and the streams of the feeds were merged before they entered the reaction unit. Samples of the product containing solvent were collected via a valve placed after the reactor for further analysis of the H_2_O_2_ concentration by external UV–vis spectroscopy, as described in [41]. The schematic representation of the experimental setup, further details about the execution of the experiments, and the data analysis can be found in the literature [23].

## 4. Conclusions

In the present study, we focused on the deposition of mono- and bimetallic Pd and Pt NPs on TiO_2_ powder; the synthesized catalysts were tested successfully for the direct synthesis of H_2_O_2_ in the liquid phase under ambient conditions.

The results presented in this work showed that both mono- and bimetallic NPs with a mean particle size between 2.0 nm and 2.8 nm could be deposited uniformly on the TiO_2_ powder via SFRD, while IWI led to larger Pd NPs with a mean PS of 7.2 nm. In contrast to the monomodal PSD for Pd and Pt NPs synthesized via SFRD, a distinct bimodal PSD was obtained for the bimetallic CAT-Pd1Pt and CAT-Pd3-IWI. In the direct synthesis of H_2_O_2_, monometallic Pd NPs on TiO_2_ powder were found to result in a clearly superior mean productivity compared to bimetallic Pd1Pt NPs and to pure Pt NPs. Furthermore, CAT-Pd1 prepared by SFRD showed a 6-fold higher mean productivity compared to the catalyst CAT-Pd3-IWI. The comparison between TiO_2_-powder catalysts and TiO_2_-coated complex stainless-steel-structure catalysts showed an up to 16 times higher mean productivity for the TiO_2_-powder catalysts.

Furthermore, results published recently showed that small (ca. 2.5 nm) Pt NPs can be deposited on TiO_2_-CeO_2_ hollow nanospheres either via SFRD or via conventional wet-chemical deposition (WCD). The catalytic properties of these nanospheres were evaluated for CO oxidation. Thereby, the SFRD catalyst showed significantly lower *T*_50_ light-off/-out temperatures than the WCD catalyst [42].

In summary, all catalysts prepared by SFRD exhibited higher catalytic activities compared to the catalysts prepared by conventional wetness impregnation methods. This was mainly due to the ability to deposit highly uniform mono- and bimetallic NPs smaller than 3 nm on various substrates via SFRD. As pointed out by Garrido et al. and Casapu et al. in more detail, this was mainly caused by a small PS and/or a narrow PSD and a higher, homogeneous dispersion of the noble metal NPs synthesized via SFRD.

Nevertheless, there is a need for a deeper understanding of the influence of the various nanoparticle preparation methods such as SFRD, IWI, and WCD on the catalyst’s properties. Besides this, the influence of particle size, size distribution, loading, and the interaction of the different metallic nanoparticles with the surface of the substrate on catalytic productivity needs to be examined more closely by scientists from different but complementary disciplines.

## Figures and Tables

**Table 1:** PS, width of the PSD, *Δ*, metal loading of mono- and bimetallic NPs on TiO_2_, and mean productivity for the different catalysts.

CAT	*x*_10_ nm	*x*_50_ nm	*x*_90_ nm	*Δ* –	Loading wt%	Mean Productivity mmol · g_metal_^−1^ · h^−1^
-Pd1	1.7	2.5	3.8	0.43	0.84	458
-Pd2	1.0	2.0	5.7	1.16	0.77	325
-Pt	1.4	2.0	4.4	0.74	1.00	179
-Pd1Pt	0.9	2.8	4.5	0.64	0.51/0.49	292
-Pd3-IWI	2.1	7.2	13.1	0.76	0.99	78

**Table 2:** Selected properties of the precursors used in this work.

	*M* g · mol^−1^	*M_metal_* g · mol^−1^	Metal Content %	Purity %
Pd(acac)_2_	304.64	106.42	34.9	99
Pd(tmhd)_2_	472.95	106.42	22.5	98
Pt(cod)me_2_	333.34	195.08	58.5	99
Pd(NO_3_)_2_ · H_2_O	266.46	106.42	39.9	95

## Data Availability

The data that support the findings of this study are available from the corresponding author upon reasonable request.
